# The effect of exercise therapy on pain, fatigue, bone function and inflammatory biomarkers individuals with rheumatoid arthritis and knee osteoarthritis: a meta-research review of randomized controlled trials

**DOI:** 10.3389/fphys.2025.1558214

**Published:** 2025-04-09

**Authors:** Xiaoting Fu, Liang Zhang, Cuijuan Wang, Jun Yue, Hang Zhu

**Affiliations:** Orthopedics Department, Hangzhou Traditional Chinese Medicine Hospital Affiliated to Zhejiang Chinese Medical University, Hangzhou City, China

**Keywords:** exercise, walk, physical activity, inflamma-tion, meta, analysis

## Abstract

**Background:**

Accumulating evidence suggested the potential role of exercise in alleviating rheumatoid arthritis (RA). However, whether exercise improves physical function (walk test, grip strength, muscle strength, joint assessments) and inflammatory biomarkers in patients with RA is unclear. This umbrella meta-analysis aimed to examine the effect of exercise in patients with RA.

**Method:**

PubMed, Scopus, Web of Science, Embase, and Cochrane Central Library databases were systematically searched for meta-analyses of randomized control trials (RCTs) to retrieve relevant studies. The effect sizes were pooled using a random-effects model, with standardized or weighted mean differences (SMDs or WMDs) and 95% confidence intervals (CIs) as summary statistics.

**Results:**

Seventeen studies were included. The improving effects of exercise on fatigue levels (SMD = −0.28, 95% CI: −0.44, −0.13), pain intensity (ES = −0.50, 95% CI: −0.87, −0.14), disease activity score in joints (DAS) (WMD = −0.54, 95% CI: −0.99, −0.09; and SMD = −0.47, 95% CI: −0.64, −0.30), and ESR (ES = −0.85, 95% CI: −1.66, −0.03) were significant. No significant impact on the hand grip, muscle strength, walk test, joints and inflammatory biomarkers was observed.

**Conclusion:**

Exercise significantly reduces fatigue, pain, DAS, and ESR in RA but shows no impact on grip strength, muscle strength, walk test, joints, or other inflammatory biomarkers. This highlights its role in symptom management rather than broad physiological changes.

## Introduction

Rheumatoid arthritis (RA), and knee osteoarthritis (OA) are chronic autoimmune disorders affecting approximately 18 million individuals worldwide (Vos, 2020). It is characterized by persistent synovial inflammation, leading to joint pain, swelling, and stiffness, which can result in significant functional impairment and reduced quality of life (Jahid et al., 2023). A hallmark of RA is the loss of immune tolerance, where the immune system erroneously targets self-antigens in the synovium. This autoimmune response leads to the activation of CD4^+^ T cells, B cells, and macrophages, resulting in the production of pro-inflammatory cytokines like tumor necrosis factor-alpha (TNF-α), interleukin-6 (IL-6), and interleukin-1 (IL-1) (Guo et al., 2018; Yasmeen et al., 2024). These cytokines perpetuate inflammation and promote the proliferation of synovial fibroblasts, contributing to pannus formation and subsequent joint destruction (Davis, 2003).

Effective RA management aims to control inflammation, prevent joint damage, and preserve functional capacity. While pharmacological agents, such as nonsteroidal anti-inflammatory drugs (NSAIDs) and biological agents, including TNF inhibitors and IL-6 receptor antagonists, are widely used to control inflammation and slow disease progression, non-pharmacological strategies play a crucial role in complementing medical therapy (Singh et al., 2015). Among these, structured exercise programs are specifically recommended to improve disease outcomes by targeting muscle strength, joint mobility, and overall physical function in RA (Health Quality Ontario, 2018). Clinical guidelines recommend exercise therapy as a primary treatment for knee QA (Holden et al., 2023). In RA, exercise interventions designed to enhance muscle support around affected joints can mitigate pain and stiffness while potentially modulating inflammatory processes (Li and Wang, 2022). A structured exercise program for people with RA typically focuses on a combination of low-impact aerobics and stretching exercises to maintain joint range of motion, and muscle strengthening exercises, all designed to alleviate pain, improve mobility, and enhance overall function (Health Quality Ontario, 2018; Ghalamsiah and Nourshahi, 2023).

Emerging evidence suggests that exercise exerts its therapeutic effects through both systemic and localized molecular pathways. Regular physical activity modulates pro-inflammatory cytokines such as IL-6 and TNF-α, while increasing anti-inflammatory cytokines like interleukin-10 (IL-10) (El-Kader et al., 2013). Additionally, mechanical loading during resistance and aerobic exercise stimulates cartilage-resident chondrocytes to produce extracellular matrix components, enhancing cartilage resilience and joint health (Sun, 2010; Smith, 2020). Neural adaptations, including increased endorphin release, further contribute to pain relief, while improvements in mitochondrial function and cardiovascular efficiency alleviate fatigue (Schwarz and Kindermann, 1992). These mechanisms highlight the multifaceted benefits of exercise in RA, and knee OA management.

Despite the promising role of exercise (Smith, 2020; Mudano et al., 2019), inconsistencies in study findings (Wu et al., 2023; Wen and Chai, 2021) and substantial heterogeneity in methodologies present challenges in drawing definitive conclusions. Variations in exercise type, frequency, duration, and intensity, coupled with differences in participant characteristics and outcome measures, have led to mixed results (Smith, 2020; Mudano et al., 2019; Wu et al., 2023; Wen and Chai, 2021). This article aims to address these gaps by conducting an umbrella meta-analysis (a comprehensive synthesis that integrates findings from previous meta-analyses) to synthesize evidence from multiple meta-analyses on the effects of different exercise modalities on pain, fatigue, and hand grip strength in individuals with RA. By systematically evaluating subgroup effects, including variations in exercise type, duration, and participant demographics, this study seeks to provide a nuanced understanding of the role of exercise in RA, and knee OA management.

## Methods

### Protocol and guidelines

In this meta-analysis, we followed PRISMA guidelines for reporting systematic reviews and meta-analyses (PRISMA) (Takkouche and Norman, 2011). It has been registered with PROSPERO under the registration number CRD42024229245.

### Search strategy

To identify relevant studies, a comprehensive search was conducted in the following databases: PubMed, Scopus, Web of Science, Embase, and Cochrane Central Library, up to November 2024. The search strategies for all databases can be found in Supplementary Material 1. To increase sensitivity, wildcard terms (e.g., “*”) were used. The search was restricted to English-language publications. Reference lists of all relevant studies were manually checked to ensure the inclusion of all eligible studies. The Kappa value obtained in this study was approximately 0.8, indicating a better inter-rater consistency compared to the Kappa values in other studies. This suggested that our evaluation criteria and methods were effective and ensured the reliability of the research results.

### Inclusion and exclusion criteria

The following PICOS criteria were applied for study selection: Population (P): Adults aged ≥18 years with RA, and knee OA: Intervention (I): exercises; Comparison (C): Placebo or control group; Outcomes (O): Walk test, pain, muscle strength, joints (joint count, joint tenderness, swollen joints, tender joint count), grip strength of both hands, fatigue, disease activity score in joints (DAS), c-reactive protein (CRP), erythrocyte sedimentation rate (ESR), interleukin 6 (IL-6), and tumour necrosis factor-alpha (TNF-α) (S): Systematic review and meta-analysis studies, providing effect sizes and corresponding confidence intervals (CI) for each outcome. Studies *in vitro* or *in vivo*, controlled clinical trials, observational studies, case reports, and quasi-experimental studies lacking adequate data for effect size calculations were excluded.

### Quality assessment

A checklist based on the Measurement Tool to Assess Systematic Reviews (AMSTAR) 2 was used by two independent reviewers to assess the methodological quality of the included studies (Shea, 2017). Discrepancies were resolved through discussion or consultation with a senior author. Studies scoring ≥7 were included in the analysis.

### Data extraction

Two independent reviewers screened the titles and abstracts and then conducted a full-text evaluation of the studies that met the eligibility criteria. Extracted data included: study characteristics (author, publication year, location), participant details (sample size and age), intervention specifics (physical exercise type, and duration), and outcomes (effect sizes (ESs) with 95% confidence intervals [CIs] for walk test, pain, muscle strength, joints, grip strength, fatigue, DAS, ESR, IL-6, TNF-α, and CRP. Disagreements were resolved through discussion with a third reviewer.

### Statistical analysis

Effect sizes [weighted mean difference (WMD) or standardized mean difference (SMD)] and corresponding CIs were pooled using random-effects models when heterogeneity was significant (*I* ^*2*^ > 50%, p < 0.1). Heterogeneity was assessed using the Cochran’s Q test and *I* ^*2*^ statistic. Subgroup analyses were performed to explore heterogeneity based on variables such as mean age, frequency, intervention duration, and type of ESs. Sensitivity analysis evaluates the reliability of the findings by removing individual studies from the analysis. Publication bias was evaluated using funnel plots (for markers with >10 included studies) and statistical tests [Begg’s (Begg and Mazumdar, 1994) and Egger’s tests (Egger et al., 1997)]. If bias was detected, a trim-and-fill method was applied. All statistical analyses were conducted using STATA software version 16.0 (Stata Corp, College Station, TX, United States), with a significance threshold of p < 0.05.

## Results

### Overview of included studies

**FIGURE 1:**
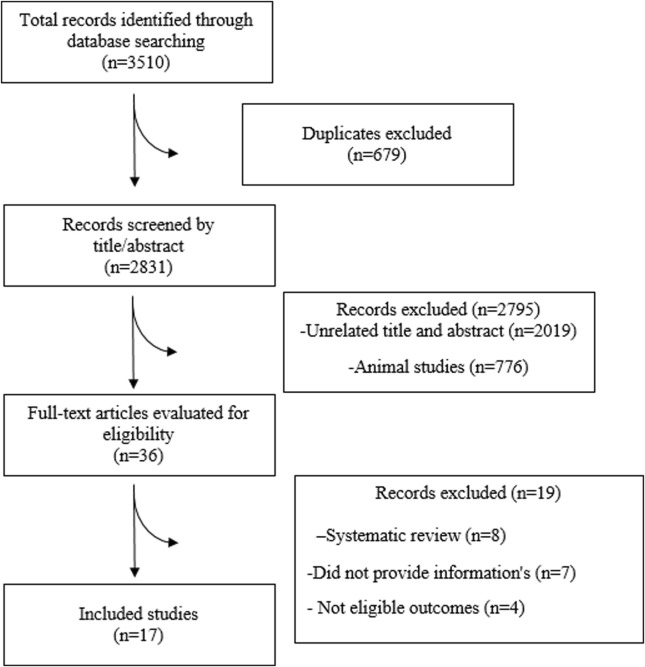
PRISMA Flowchart diagram.

Of the initial 3,510 records retrieved from the electronic databases, 679 studies were identified as duplicates and excluded, while 2,831 publications were ruled out after reviewing titles and abstracts. The remaining 36 studies underwent full-text screening, during which 19 were excluded based on criteria specified in the PRISMA flowchart (Figure 1). Ultimately, 17 meta-analyses met the eligibility criteria and were included in the umbrella meta-analysis. The basic characteristics of the included articles are shown in Table 1. The publication years of these studies ranged from 2004 to 2023. The exercise duration varies from 15 to 50 min, and the frequency of exercise sessions ranges from one to four. The number of participants ranged from 206 to 2,405, with ages varying between 47 and 58 years. Most studies have used aerobic exercise, and some have combined aerobic with resistance exercise.

**TABLE 1:** The basic characteristics of the included articles.

Author names and years	Sample size	Gender/Age (year)	Health conditions	Type of effect size and results	Physical exercise	Duration (min)/Frequency
Baillet et al. (2012)	547	Both/51.5	RA	↓Walk; WMD: 1.9 (−2.95, −0.85) ↓ESR; WMD: 5.17 (−8.77, −1.58) ↓Joint count; WMD: 5.39 (−9, −1.72) ↑Grip strength; WMD: 24.4 (12.3, 40.5) ↓Pain; WMD: 4.13 (−11.0, −2.71)	Resistance exercises	20/2
Han et al. (2004)	206	Both/55	RA	↔Walk; WMD: 0.35 (−1.14, 1.84) ↔Tender joints; WMD: 0.83 (−3.3, 1.64) ↔Swollen joints; WMD: 2.45 (−0.45, 5.63) ↔Grip strength; WMD: 0.08 (−0.26, 0.1)	Aerobic Exercise	30/3
Wen and Chai (2021)	512	Both/56.5	RA	↓Walk; SMD: 0.64 (−0.99, −0.28) ↓ESR; SMD: 0.86 (−1.65, −0.07) ↔Pain; SMD: 0.61 (−1.49, 0.27)	Resistance exercises	30/3
Wu et al. (2023)	351	Both/53.5	RA	↔Walk; WMD: 0.17 (−1.06, 1.4) ↔Tender joints; WMD: 0.01 (−0.32, 0.29) ↔Swollen joints; WMD: 0.5 (−2.09, 3.1) ↔Tender joints; WMD: 0.41 (−5.18, 6.01) ↔Grip strength; WMD: 0.08 (−0.26, 0.1) ↔Pain; WMD: 0.88 (−1.99, 0.23)	Aerobic Exercise	50/2
Mudano et al. (2019)	326	Both/54	RA	↔Walk; WMD: 0.17 (−1.06, 1.4) ↔Tender joints; WMD: 0.74 (−3.12, 1.65) ↔Swollen joints; WMD: 2.59 (−0.18, 5.35) ↔Grip strength; WMD: 3.12 (−9.99, 3.76) ↓Pain; SMD: 0.95 (−1.41, −0.49)	Aerobic Exercise	22/3
Sobue et al. (2022)	1343	Both/58.5	RA	↔DAS; WMD: 0.52 (−1.25, 0.21) ↓Pain; SMD: 2.04 (−3.77, −0.32)	Aerobic + resistance exercise	20/3
Liu et al. (2023)	483	Both/56	RA	↓Grip strength; WMD: 0.21 (−0.39, −0.03) ↓Pain; WMD: 1.22 (−1.76, −0.67)	Aerobic + resistance exercise	15/1
Sezgin et al. (2023)	983	Both/50	RA	↑Pain; SMD: 0.22 (0.06, 0.38)	Aerobic + resistance exercise	30/4
Ye et al. (2020)	840	Both/47	RA	↓DAS; SMD: 0.39 (−0.68, −0.1) ↔CRP; SMD: 0.29 (−0.81, 0.23) ↔ESR; SMD: 0.29 (−1.13, 0.55) ↔IL-6; SMD: 0.55 (−1.77, 0.08) ↔TNF-a; SMD: 0.59 (−1.23, 0.06) ↔Tender joints; SMD: 0.39 (−1.38,0.61) ↔Swollen joints; SMD: 0.88 (−2.78,1.02) ↑Grip strength; WMD: 1.3 0.47, 2.13 ↔Pain; SMD: 0.86(−1.93, 0.22)	Aerobic Exercise	24/2
Andrea Cortés-Ladino et al. (2023)	600	Both/49	RA	↓DAS; SMD: 0.51 (−0.71, −0.3)	Aerobic Exercise	35/3
Ye et al. (2022)	992	Both/58	RA	↔DAS; WMD: 0.55 (−1.12, 0.01) ↔CRP; WMD: 1.08 (−2.2, 0.05) ↔ESR; SMD: 0.76 (−1.67, 0.14) ↔Joint count; SMD: 0.59 (−1.26, 0.07) ↔Tender joints; SMD: 0.19 (−0.48, 0.1) ↔Pain; SMD: 0.46(−0.9, 0.76)	Aerobic Exercise	45/3
Baillet et al. (2010)	1,040	Both/56	RA	↔Joint count; SMD: 0.14 (−0.05, 0.33) ↑Pain; SMD: 0.31 (0.06, 0.55)	Aerobic Exercise	45/2
Hurkmans et al. (2009)	575	Both/52	RA	↔Muscle strength; SMD: 0.38 (−1.67, 0.9) ↑Muscle strength; SMD: 0.47 (0.01, 0.93) ↔Muscle strength; SMD: 0.38 (−1.27, 0.51) ↔Pain; SMD: 0.27 (−0.79, 0.26) ↔Pain; SMD: 0.53 (−1.09, 0.04) ↔Pain; SMD: 0.06 (−0.43, 0.54)	Aerobic Exercise Aerobic + resistance exercise Water-based aerobic Aerobic Exercise Aerobic + resistance exercise Water-based aerobic	45/2 50/2 30/2 45/2 50/2 30/2
Williams et al. (2018)	841	Both/57	RA	↑Grip strength; WMD: 0.45 0.12, 0.79 ↓Pain; WMD: 15.61 (−28.2, −2.93)	Aerobic + resistance exercise	25/2
Rongen-van Dartel et al. (2015)	298	Both/51	RA	↓Fatigue; SMD: 0.22 (−0.43, −0.01)	Aerobic Exercise	15/2
Kelley et al. (2018)	1,286	Both/55	RA	↓Fatigue; SMD: 0.2 (−0.34, −0.06)	Aerobic Exercise	20/2
Runge et al. (2023)	1,263	Both/52	RA	↓Fatigue; SMD: 0.45 (−0.64, −0.25)	Aerobic + resistance exercise	15/3

### Quality of methodology

Regarding the quality of studies based on the AMSTAR-2 criteria, six studies were rated as high quality, and 11 as moderate. The quality of assessment is shown in Table 2.

**TABLE 2:** Results of assess the methodological quality of meta-analysis.

Study	Q1^1^	Q2	Q3	Q4	Q5	Q6	Q7	Q8	Q9	Q10	Q11	Q12	Q13	Q14	Q15	Q16	Quality assessment
Baillet et al. (2012)	No	Partial Yes	Yes	Partial Yes	Yes	Yes	Yes	Yes	No	Yes	Yes	Yes	No	Yes	No	Yes	Moderate
Han et al. (2004)	No	Partial Yes	Yes	Partial Yes	Yes	Yes	Yes	Yes	No	Yes	No	No	Yes	No	Yes	Yes	Moderate
Wen and Chai (2021)	No	Partial Yes	Yes	Partial Yes	Yes	Yes	Partial Yes	Yes	Yes	Yes	Yes	Yes	Yes	Yes	Yes	No	High
Wu et al. (2023)	No	Partial Yes	Yes	Partial Yes	Yes	Yes	Yes	Yes	Yes	Yes	Yes	Yes	Yes	Yes	No	Yes	High
Mudano et al. (2019)	No	Partial Yes	Yes	Yes	Yes	No	No	Yes	Yes	Yes	No	Yes	Yes	Yes	Yes	Yes	Moderate
Sobue et al. (2022)	No	Partial Yes	Yes	Yes	No	Yes	Yes	Yes	Yes	No	Yes	No	Yes	No	Yes	Yes	Moderate
Liu et al. (2023)	No	Yes	Yes	Partial Yes	Yes	Yes	Yes	Yes	Yes	Yes	Yes	No	Yes	Yes	Yes	Yes	High
Sezgin et al. (2023)	No	Partial Yes	Yes	Partial Yes	Yes	Yes	Yes	Yes	Yes	Yes	Yes	Yes	Yes	Yes	Yes	Yes	High
Ye et al. (2020)	No	Partial Yes	Yes	Partial Yes	No	Yes	No	Yes	Yes	Yes	Yes	Yes	Yes	Yes	Yes	Yes	Moderate
Andrea Cortés-Ladino et al. (2023)	Yes	Yes	Yes	Yes	Yes	Yes	Yes	Yes	Yes	Yes	Yes	Yes	Yes	Yes	Yes	Yes	High
Ye et al. (2022)	No	Yes	Yes	Partial Yes	Yes	Yes	Yes	Yes	Yes	Yes	Yes	Yes	Yes	Yes	No	No	Moderate
Baillet et al. (2010)	No	Yes	Yes	Partial Yes	Yes	Yes	Yes	No	No	Yes	No	Yes	Yes	No	Yes	Yes	Moderate
Hurkman et al., 2009	No	Yes	Yes	Partial Yes	Yes	Yes	Yes	Yes	Yes	Yes	Yes	No	No	No	Yes	No	Moderate
William et al. (2018)	No	Partial Yes	Yes	Partial Yes	Yes	Yes	Yes	Yes	Yes	Yes	Yes	Yes	Yes	Yes	Yes	Yes	High
Rongen-van Dartel et al. (2015)	No	Yes	Yes	Yes	Yes	Yes	Yes	Yes	No	Yes	Yes	Yes	Yes	No	No	Yes	Moderate
Kelley et al. (2018)	No	Partial Yes	Yes	Yes	Yes	Yes	No	Yes	Yes	Yes	Yes	Yes	Yes	No	No	Yes	Moderate
Runge et al. (2023)	No	Yes	Yes	Partial Yes	Yes	Yes	Yes	Yes	Yes	Yes	Yes	Yes	Yes	Yes	No	No	Moderate

### Effect of exercise on hand grip and muscle strength

**FIGURE 2:**
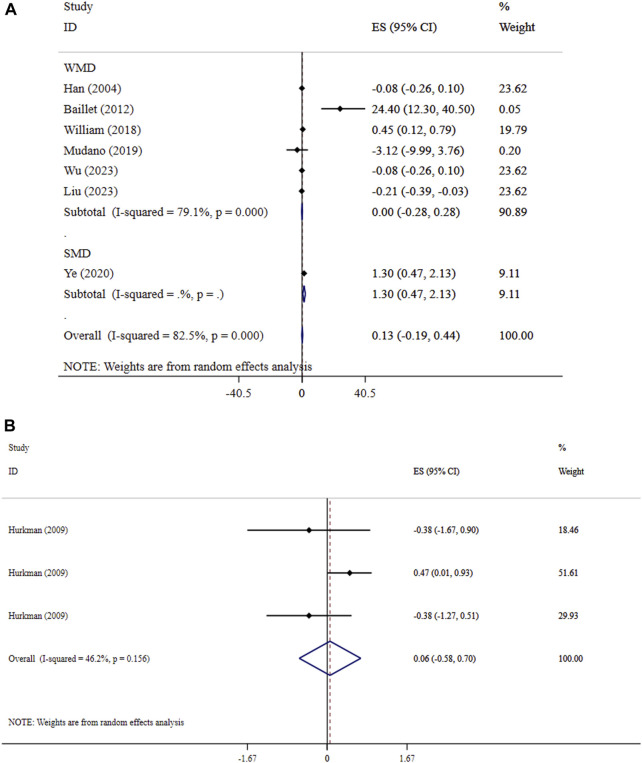
Forest plot detailing mean difference and 95% confidence intervals (CIs), the effects of exercise on hand grip and **(A)**, and muscle strength **(B)**.

Our findings revealed that exercise did not significantly improve hand grip (ES = 0.13, 95% CI: −0.19, 0.44; P = 0.431; *I* ^*2*^ = 82.5%, p < 0.001) (Figure 2A) and muscle strength (SMD = 0.06, 95% CI: −0.58, 0.70; P = 0.858; *I* ^*2*^ = 46.2%, p = 0.156) (Figure 2B). The non-significant outcomes from Begg’s tests confirm the reliability of the results from the meta-analyses (P > 0.05).

### Effect of exercise on joints and walk test

**FIGURE 3:**
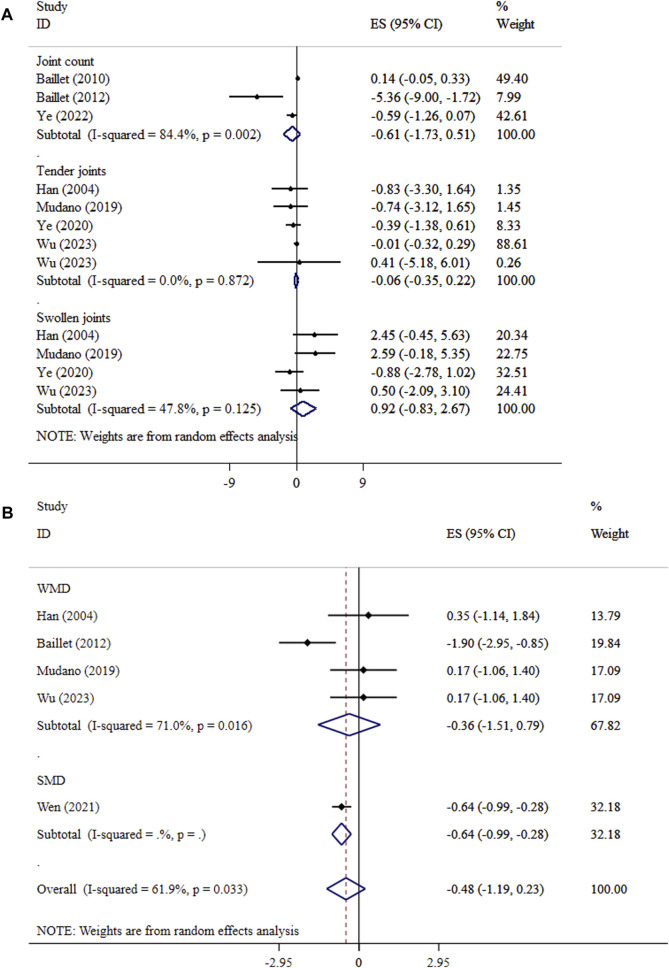
Forest plot detailing mean difference and 95% confidence intervals (CIs), the effects of exercise on joints and **(A)**, and walk test **(B)**.

Exercise did not lead to significant effects on joint count (ES = −0.61, 95% CI: −1.73, 0.51; P = 0.286; *I* ^*2*^ = 84.4%, p = 0.002), tender joints (ES = −0.06, 95% CI: −0.35, 0.22; P = 0.671; *I* ^*2*^ = 0.0%, P = 0.872), swollen joints (ES = 0.92, 95% CI: −0.83, 2.67; P = 0.301; *I* ^*2*^ = 47.8%, p = 0.125), and walk test (ES = −0.48, 95% CI: −1.19, 0.23; p = 0.189; *I* ^*2*^ = 61.9%, p = 0.033) in patients with RA (Figures 3A, B). The sensitivity analysis results supported the outcome’s validity (p < 0.05). The results of Begg’s tests did not detect publication bias (P > 0.05).

### Effect of exercise on fatigue

**FIGURE 4:**
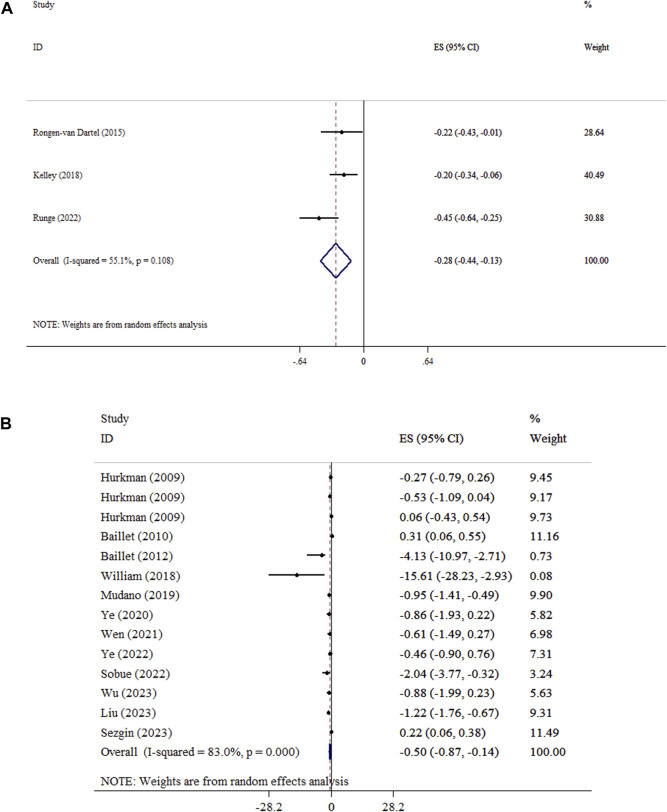
Forest plot detailing mean difference and 95% confidence intervals (CIs), the effects of exercise on fatigue and **(A)**, and pain **(B)**.

The improving effect of exercise on fatigue levels was significant (SMD = −0.28, 95% CI: −0.44, −0.13; p < 0.001; *I* ^*2*^ = 55.1%, p = 0.108) (Figure 4A). The results of the SMD analysis and the sensitivity testing were consistent (P < 0.05). Subgroup analyses showed that the largest decreases in fatigue were observed in aerobic with resistance exercise (Table 3). Begg’s tests showed no statistical evidence of publication bias (P > 0.05).

**TABLE 3:** Subgroup analyses for the effects of exercise on health outcomes.

	Effect size, *n*	ES (95% CI)^1^	*I* ^2^ (%)^3^	P-heterogeneity^4^
Pain				
Overall	14	−0.50 (−0.87, −0.14)	83.0	<0.001
Duration (min)				
<30	7	−1.09 (−1.59, −0.60)	40.7	0.119
≥30	7	−0.04 (−0.30, 0.22)	62.3	0.014
Type of exercise				
Resistance exercises	2	−1.77 (−5.01, 1.47)	65.2	0.102
Aerobic Exercise	6	−0.45 (−1.02, 0.11)	82.6	<0.001
Aerobic + resistance	5	−0.81 (−1.76, 0.14)	90.2	<0.001
Water-based aerobic	1	0.06 (−0.43, 0.55)	—	—
Age(year)				
≤55	7	−0.38 (−0.85, 0.09)	82.5	<0.001
>55	7	−0.76 (−1.56, 0.04)	85.5	<0.001
Frequency (week)				
<3	7	−0.77 (−1.58, 0.03)	85.9	<0.001
≥3	7	−0.39 (−0.86, 0.07)	81.8	<0.001
Type of effect size				
WMD	4	−1.48 (−2.77, −0.18)	58.6	0.065
SMD	10	−0.31 (−0.64, 0.02)	79.7	<0.001
Fatigue				
Overall	3	−0.28 (−0.44, −0.13)	55.1	0.108
Type of exercise				
Aerobic Exercise	2	−0.21 (−0.32, −0.09)	0.0	0.877
Aerobic + resistance	1	−0.45 (−0.64, −0.25)	—	—
Hand grip				
Overall	7	0.13 (−0.19, 0.44)	82.5	<0.001
Age (year)				
≤55	3	4.15 (−5.11, 13.40)	83.8	<0.001
>55	4	0.19 (−0.18, 0.57)	86.3	<0.001
Duration (min)				
<30	5	0.47 (−0.36, 1.30)	88.2	<0.001
≥30	2	−0.08 (−0.21, 0.05)	0.0	0.810
Type of exercise				
Resistance exercises	1	24.40 (10.30, 38.50)	—	—
Aerobic + resistance	2	0.10 (−0.54, 0.75)	91.4	<0.001
Aerobic Exercise	4	0.09 (−0.26, 0.44)	73.1	0.011

### Effect of exercise on pain

Our results showed that exercise reduced pain intensity in patients with RA (ES = −0.50, 95% CI: −0.87, −0.14; P=0.007), with high heterogeneity (*I* ^*2*^ = 83.0%, P<0.001) (Figure 4B), and in patients with knee OA (SMD = −0.49, 95% CI: −0.61, −0.37; P<0.001). The conclusions remained stable, as verified by the sensitivity analysis. Subgroup analysis showed that exercise led to the greatest reduction in pain intensity in duration <30 min, and ES type (WMD analysis) (Table 3). Findings from Egger’s and Begg’s tests and visual inspection of the funnel plot confirm the absence of significant publication bias (P > 0.05) (Supplementary Figure 1).

### Effect of exercise on DAS

**FIGURE 5:**
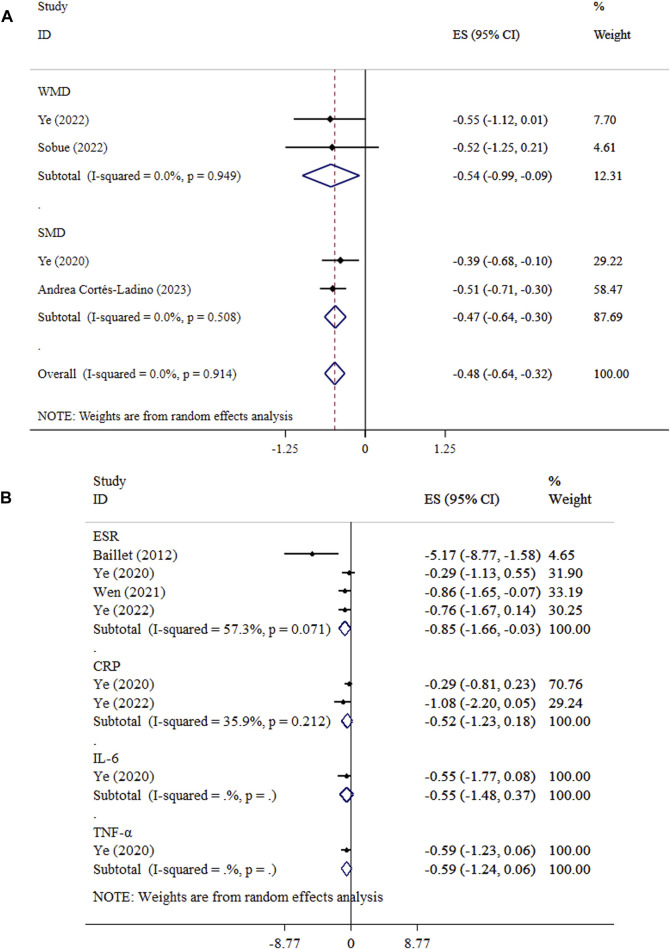
Forest plot detailing mean difference and 95% confidence intervals (CIs), the effects of exercise on DAS and **(A)**, and inflammatory biomarkers **(B)**.

Exercise significantly decreased DAS levels in both WMD and SMD analyses. The WMD analysis demonstrated an overall effect size of −0.54 (95% CI: −0.99, −0.09; P = 0.018) with low heterogeneity (*I* ^*2*^ = 0.0%, P = 0.949). Similarly, the SMD analysis showed a significant decrease (SMD = −0.47, 95% CI: −0.64, −0.30; P < 0.001; *I* ^*2*^ = 0.0%, P = 0.508) (Figure 5A). Sensitivity analysis approved the robustness of findings in both WMD and SMD metrics. Publication bias was not detected using Begg’s test. (P > 0.05).

### Effect of exercise on inflammatory biomarkers

The effect of exercise on CRP (ES = −0.52, 95% CI: −1.23, 0.18; P = 0.147; *I* ^*2*^ = 35.9%, P = 0.212), IL-6 (ES = −0.55, 95% CI: −1.48, 0.37; P = 0.244), and TNF-α (ES = −0.59, 95% CI: −1.24, 0.06; P = 0.073) levels did not reach statistical significance (Figure 5B). Begg’s tests indicated no evidence of publication bias (P > 0.05). Exercise led to a significant reduction in ESR (ES = −0.85, 95% CI: −1.66, −0.03; P = 0.042; *I* ^*2*^ = 5.3%, P = 0.071).

## Discussion

This study evaluated the impact of exercise interventions on various health outcomes in patients with RA. The analysis revealed that exercise did not significantly improve hand grip strength or overall muscle strength, as well as joint count metrics, including total joint count, tender joints, and swollen joints. Performance in walk tests also did not show significant enhancement. Conversely, exercise in both aerobic and resistance type interventions led to a significant reduction in fatigue levels. WMD analysis showed that pain intensity decreased significantly following exercise, particularly in interventions lasting less than 30 min per session in patients with RA, and knee OA. However, results on pain must be interpreted with precaution due to high heterogeneity, as well as the non-significant effects in other subgroups. Regarding disease activity, exercise significantly lowered DAS levels. Inflammatory biomarkers such as CRP, IL-6, and TNF-α did not show significant changes post-exercise. However, ESR demonstrated a significant reduction. The significant results on pain, fatigue, and DAS were not clinically important (Ward et al., 2015). Defining an exact clinically important difference for ESR is challenging due to its variability and sensitivity to numerous factors beyond RA activity (Ward, 2004). Consequently, ESR changes are often interpreted in conjunction with other clinical assessments. Therefore, exercise can only be considered as an adjutant therapy approach or preventive strategy in managing RA patients (Tahira, 2023; Faraziani and Eken, 2024).

The findings suggest that while exercise interventions may not significantly enhance hand grip strength, overall muscle strength, joint counts, or walk test performance in patients with RA, they are effective in reducing fatigue, pain intensity, and disease activity. The lack of improvement in muscle strength and joint metrics could be attributed to the heterogeneity of exercise protocols, variations in disease severity among participants, and the relatively short duration of some interventions.

The observed small but significant reduction in fatigue with aerobic exercise may be attributed to its positive effects on cardiovascular fitness (Patel et al., 2017), muscle endurance (Markov et al., 2022), and overall systemic inflammation (Al-Jiffri and Abd El-Kader, 2021), all of which are key contributors to fatigue in individuals with RA. Aerobic exercise enhances oxygen delivery to tissues (Joyner and Casey, 2015), reduces lactate buildup (Spurway, 1992), and improves mitochondrial function (Porter et al., 2015), which collectively increase energy efficiency and reduce perceived fatigue. Additionally, regular aerobic activity may modulate inflammatory cytokines, potentially alleviating the fatigue associated with systemic inflammation in RA (Al-Jiffri and Abd El-Kader, 2021). Aerobic exercise training is more effective than resistance exercise in modulation of inflammatory cytokines (Al-Jiffri and Abd El-Kader, 2021). Engaging in resistance training can enhance proteins that are involved in muscle remodeling and angiogenesis, leading to relieving fatigue (Englund et al., 2022). However, resistance training, while beneficial for building muscle strength and functional capacity, may not directly target the physiological mechanisms underlying fatigue to the same extent as aerobic exercise (Schroeder et al., 2019). Some metabolites including thromboxane, prostaglandins, bradykinin and purinergic type 2X receptors, and ion channels in response to resistance exercise may be involved in fatigue induced by resistance training (Zając et al., 2015). However, combining aerobic and resistance exercises could not dilute the specific benefits of aerobic on fatigue.

Duration of exercise sessions emerged as an important modifier of pain outcomes. Interventions with sessions lasting less than 30 min were associated with a stronger reduction in pain, possibly due to better adherence or reduced risk of exacerbating joint pain and fatigue. However, the high heterogeneity of pooled analysis indicates variability in responses, underscoring the need for personalized exercise programs tailored to individual characteristics. Exercise stimulates the production of endorphins, as the natural painkillers and mood elevators (Lima et al., 2017). Moreover, engaging in exercise can lead to a temporary reduction in pain sensitivity, known as exercise-induced hypoalgesia (EIH) (Rice et al., 2019). In addition, exercise influences pain processing pathways, potentially altering nociceptive, neuropathic, and psychosocial pain mechanisms (Chimenti et al., 2018). However, the analgesic effects of exercise can vary, especially in individuals with chronic pain, where central analgesic systems may respond differently (Nijs et al., 2012).

The discrepancy in the significance of exercise effects on pain between WMD and SMD analyses can be attributed to the different ways these effect size metrics handle variability in the outcome measures. WMD directly uses the raw mean differences in pain scores between intervention and control groups, maintaining the original scale of measurement (Andrade, 2020). This makes WMD particularly sensitive to absolute changes in pain levels, especially in studies using the same or similar pain scales. In this case, WMD might have captured a consistent reduction in pain across studies that used comparable measurement tools, leading to a significant result. On the other hand, SMD standardizes the effect size by dividing the mean difference by the pooled standard deviation, allowing comparison across studies that use different pain scales. This standardization reduces the impact of absolute changes and emphasizes the relative magnitude of the effect in the context of variability within and between studies. If there is substantial heterogeneity in study designs, population characteristics, or baseline pain levels, SMD can dilute the observed effect, leading to a non-significant result (Andrade, 2020).

The reduction in DAS signifies that exercise contributes to overall disease activity attenuation, possibly by modulating inflammatory processes and improving physical function. The non-significant changes in CRP, IL-6, and TNF-α levels suggest that while exercise improves clinical outcomes, its impact on systemic inflammation markers may be limited or require longer intervention periods to become evident. The significant decrease in ESR indicates a potential reduction in inflammation, as ESR is a general marker of inflammatory activity (Alende-Castro et al., 2019). Moreover, the limited measurement of inflammatory biomarkers (CRP in only two studies, IL-6 and TNF-α in just one) may have introduced bias, potentially contributing to the observed lack of significant results, thereby affecting the robustness and generalizability of the findings. Exercise potentially improves pain and fatigue through a reduction in systemic inflammation, mediated by decreased levels of pro-inflammatory cytokines such as IL-6, TNF-α, and CRP (Andrade, 2020). Increased production of anti-inflammatory cytokines like IL-10 may further contribute to symptom relief (Małkowska and Sawczuk, 2023). In the context of joint health, mechanical loading during resistance and aerobic exercise may stimulate chondrocytes to produce extracellular matrix components, thereby improving cartilage resilience (Leong and Sun, 2014). Emerging evidence suggests that exercise may also influence epigenetic regulation of inflammatory genes, thereby exerting long-term anti-inflammatory effects (Plaza-Diaz et al., 2022).

This umbrella meta-analysis has several limitations that must be considered. First, the high degree of heterogeneity (I^2^ > 60% in most subgroups) across studies, particularly in terms of exercise duration and type, as well as age, limits the generalizability of the findings. Variability in reporting standards and the use of different effect size metrics, such as WMD and SMD, further complicates comparisons. Additionally, subgroup analyses were constrained by the availability of data, resulting in small sample sizes for specific exercise types. Limited short intervention periods of included studies limits the generalizability of the findings. Another notable limitation is the reliance on self-reported outcomes, such as pain and fatigue, which are subjective and susceptible to bias. However, the strengths of this study include its comprehensive scope, the integration of multiple meta-analyses, and the systematic exploration of key moderators such as age, and exercise frequency. By synthesizing evidence from diverse populations, this review provides a more holistic understanding of exercise interventions in RA management.

Future research should focus on standardizing methodologies in exercise trials to reduce heterogeneity and enhance comparability across studies. Investigations into novel exercise regimens, such as high-intensity interval training or tailored interventions for specific subpopulations, are warranted. Longitudinal studies with longer follow-up periods are needed to assess the sustainability of benefits. Moreover, future trials should include objective biomarkers to complement subjective measures, offering a more precise evaluation of exercise effects. Incorporating molecular and mechanistic analyses in these trials could elucidate the pathways through which exercise modulates RA outcomes, paving the way for personalized exercise prescriptions. Moreover, the combination of dietary factors and nutritional approaches with physical activity in future studies can provide innovative strategies for managing RA. This integration can synergize the anti-inflammatory and antioxidant effects of a healthy diet with the benefits of improved muscle strength, flexibility, and pain reduction achieved through exercise.

## Conclusion

This umbrella meta-analysis underscores the beneficial role of exercise in managing RA, and knee OA, particularly in reducing pain, fatigue, disease activity, and ESR. Future research should focus on personalized approaches, novel regimens, and objective biomarkers to refine non-pharmacological strategies for RA, and knee OA management.

## Data Availability

The datasets presented in this study can be found in online repositories. The names of the repository/repositories and accession number(s) can be found in the article/Supplementary Material.
