# Supplementary material for: The effect of exercise therapy on pain, fatigue, bone function and inflammatory biomarkers individuals with rheumatoid arthritis and knee osteoarthritis: a meta-research review of randomized controlled trials
Source: Front Physiol. 2025 Apr 9;16:1558214. doi: 10.3389/fphys.2025.1558214 (PMC12014597; doi:10.3389/fphys.2025.1558214)
Supplement: Supplementary file 1 [file Table1.docx]

| **Author names and years** | **Sample size** | **Gender/ Age (year)** | **Health conditions** | **Type of effect size and results** | **Physical exercise** | **Duration (min)/ Frequency** |
| --- | --- | --- | --- | --- | --- | --- |
| Baillet et al.2012 | 547 | Both/ 51.5 | RA | ↓Walk; WMD: -1.9(-2.95, -0.85)  ↓ESR; WMD: -5.17(-8.77, -1.58)  ↓Joint count; WMD: -5.39(-9, -1.72)  ↑Grip strength; WMD: 24.4(12.3, 40.5)  ↓Pain; WMD: -4.13 (-11.0, -2.71) | Resistance exercises | 20/2 |
| Han et al.2004 | 206 | Both/ 55 | RA | ↔Walk; WMD: 0.35 (-1.14,1.84)  ↔Tender joints; WMD: -0.83 (-3.3,1.64)  ↔Swollen joints; WMD: 2.45 (-0.45,5.63)  ↔Grip strength; WMD: -0.08 (-0.26,0.1) | Aerobic Exercise | 30/3 |
| Wen et al.2021 | 512 | Both/ 56.5 | RA | ↓Walk; SMD: -0.64 (-0.99, -0.28)  ↓ESR; SMD: -0.86 (-1.65, -0.07)  ↔Pain; SMD: -0.61(-1.49, 0.27) | Resistance exercises | 30/3 |
| Wu et al.2023 | 351 | Both/ 53.5 | RA | ↔Walk; WMD: 0.17 (-1.06,1.4)  ↔Tender joints; WMD: -0.01 (-0.32,0.29)  ↔Swollen joints; WMD: 0.5 (-2.09,3.1)  ↔Tender joints; WMD: 0.41 (-5.18,6.01)  ↔Grip strength; WMD: -0.08 (-0.26, 0.1)  ↔Pain; WMD: -0.88(-1.99, 0.23) | Aerobic Exercise | 50/2 |
| Mudano et al.2019 | 326 | Both/ 54 | RA | ↔Walk; WMD: 0.17 (-1.06,1.4)  ↔Tender joints; WMD: -0.74 (-3.12,1.65)  ↔Swollen joints; WMD: 2.59 (-0.18,5.35)  ↔Grip strength; WMD: -3.12 (-9.99, 3.76)  ↓Pain; SMD: -0.95(-1.41, -0.49) | Aerobic Exercise | 22/3 |
| Sobue et al.2022 | 1343 | Both/ 58.5 | RA | ↔DAS; WMD: -0.52(-1.25, 0.21)  ↓Pain; SMD: -2.04(-3.77, -0.32) | Aerobic + resistance exercise | 20/3 |
| Liu et al.2023 | 483 | Both/ 56 | RA | ↓Grip strength; WMD: -0.21 (-0.39, -0.03)  ↓Pain; WMD: -1.22(-1.76, -0.67) | Aerobic + resistance exercise | 15/1 |
| Sezgin et al.2023 | 983 | Both/ 50 | RA | ↑Pain; SMD: 0.22(0.06, 0.38) | Aerobic + resistance exercise | 30/4 |
| Ye et al.2020 | 840 | Both/ 47 | RA | ↓DAS; SMD: -0.39(-0.68, -0.1)  ↔CRP; SMD: -0.29(-0.81, 0.23)  ↔ESR; SMD: -0.29(-1.13, 0.55)  ↔IL-6; SMD: -0.55(-1.77, 0.08)  ↔TNF-a; SMD: -0.59(-1.23, 0.06)  ↔Tender joints; SMD: -0.39 (-1.38,0.61)  ↔Swollen joints; SMD: -0.88 (-2.78,1.02)  ↑Grip strength; WMD: 1.3 0.47, 2.13)  ↔Pain; SMD: -0.86(-1.93, 0.22) | Aerobic Exercise | 24/2 |
| Andrea Cortés-Ladino et al.2023 | 600 | Both/ 49 | RA | ↓DAS; SMD: -0.51(-0.71, -0.3) | Aerobic Exercise | 35/3 |
| Ye et al.2022 | 992 | Both/ 58 | RA | ↔DAS; WMD: -0.55(-1.12, 0.01)  ↔CRP; WMD: -1.08(-2.2, 0.05)  ↔ESR; SMD: -0.76(-1.67, 0.14)  ↔Joint count; SMD: -0.59(-1.26, 0.07)  ↔Tender joints; SMD: -0.19 (-0.48,0.1)  ↔Pain; SMD: -0.46(-0.9, 0.76) | Aerobic Exercise | 45/3 |
| Baillet et al.2010 | 1040 | Both/ 56 | RA | ↔Joint count; SMD: 0.14(-0.05, 0.33)  ↑Pain; SMD: 0.31(0.06, 0.55) | Aerobic Exercise | 45/2 |
| Hurkman et al.2009 | 575 | Both/ 52 | RA | ↔Muscle strength; SMD: -0.38 (-1.67, 0.9)  ↑Muscle strength; SMD: 0.47 (0.01, 0.93)  ↔Muscle strength; SMD: -0.38 (-1.27, 0.51)  ↔Pain; SMD: -0.27(-0.79, 0.26)  ↔Pain; SMD: -0.53(-1.09, 0.04)  ↔Pain; SMD: 0.06(-0.43, 0.54) | Aerobic Exercise  Aerobic + resistance exercise  Water-based aerobic  Aerobic Exercise  Aerobic + resistance exercise  Water-based aerobic | 45/2  50/2  30/2  45/2  50/2  30/2 |
| William et al.2018 | 841 | Both/ 57 | RA | ↑Grip strength; WMD: 0.45 0.12, 0.79)  ↓Pain; WMD: -15.61(-28.2, -2.93) | Aerobic + resistance exercise | 25/2 |
| Rongen-van Dartel et al.2015 | 298 | Both/ 51 | RA | ↓Fatigue; SMD: -0.22(-0.43, -0.01) | Aerobic Exercise | 15/2 |
| Kelley et al.2018 | 1286 | Both/ 55 | RA | ↓Fatigue; SMD: -0.2 (-0.34, -0.06) | Aerobic Exercise | 20/2 |
| Runge et al.2022 | 1263 | Both/ 52 | RA | ↓Fatigue; SMD: -0.45 (-0.64, -0.25) | Aerobic + resistance exercise | 15/3 |

**Table 1**. The basic characteristics of the included articles
