# Supplementary material for: The effect of exercise therapy on pain, fatigue, bone function and inflammatory biomarkers individuals with rheumatoid arthritis and knee osteoarthritis: a meta-research review of randomized controlled trials
Source: Front Physiol. 2025 Apr 9;16:1558214. doi: 10.3389/fphys.2025.1558214 (PMC12014597; doi:10.3389/fphys.2025.1558214)
Supplement: Supplementary file 2 [file Table2.docx]

| Study | **Q1^1^** | **Q2** | **Q3** | **Q4** | **Q5** | **Q6** | **Q7** | **Q8** | **Q9** | **Q10** | **Q11** | **Q12** | **Q13** | **Q14** | **Q15** | **Q16** | **Quality assessment** |
| --- | --- | --- | --- | --- | --- | --- | --- | --- | --- | --- | --- | --- | --- | --- | --- | --- | --- |
| Baillet et al.2012 | No | Partial Yes | Yes | Partial Yes | Yes | Yes | Yes | Yes | No | Yes | Yes | Yes | No | Yes | No | Yes | Moderate |
| Han et al.2004 | No | Partial Yes | Yes | Partial Yes | Yes | Yes | Yes | Yes | No | Yes | No | No | Yes | No | Yes | Yes | Moderate |
| Wen et al.2021 | No | Partial Yes | Yes | Partial Yes | Yes | Yes | Partial Yes | Yes | Yes | Yes | Yes | Yes | Yes | Yes | Yes | No | High |
| Wu et al.2023 | No | Partial Yes | Yes | Partial Yes | Yes | Yes | Yes | Yes | Yes | Yes | Yes | Yes | Yes | Yes | No | Yes | High |
| Mudano et al.2019 | No | Partial Yes | Yes | Yes | Yes | No | No | Yes | Yes | Yes | No | Yes | Yes | Yes | Yes | Yes | Moderate |
| Sobue et al.2022 | No | Partial Yes | Yes | Yes | No | Yes | Yes | Yes | Yes | No | Yes | No | Yes | No | Yes | Yes | Moderate |
| Liu et al.2023 | No | Yes | Yes | Partial Yes | Yes | Yes | Yes | Yes | Yes | Yes | Yes | No | Yes | Yes | Yes | Yes | High |
| Sezgin et al.2023 | No | Partial Yes | Yes | Partial Yes | Yes | Yes | Yes | Yes | Yes | Yes | Yes | Yes | Yes | Yes | Yes | Yes | High |
| Ye et al.2020 | No | Partial Yes | Yes | Partial Yes | No | Yes | No | Yes | Yes | Yes | Yes | Yes | Yes | Yes | Yes | Yes | Moderate |
| Andrea Cortés-Ladino et al.2023 | Yes | Yes | Yes | Yes | Yes | Yes | Yes | Yes | Yes | Yes | Yes | Yes | Yes | Yes | Yes | Yes | High |
| Ye et al.2022 | No | Yes | Yes | Partial Yes | Yes | Yes | Yes | Yes | Yes | Yes | Yes | Yes | Yes | Yes | No | No | Moderate |
| Baillet et al.2010 | No | Yes | Yes | Partial Yes | Yes | Yes | Yes | No | No | Yes | No | Yes | Yes | No | Yes | Yes | Moderate |
| Hurkman et al.2009 | No | Yes | Yes | Partial Yes | Yes | Yes | Yes | Yes | Yes | Yes | Yes | No | No | No | Yes | No | Moderate |
| William et al.2018 | No | Partial Yes | Yes | Partial Yes | Yes | Yes | Yes | Yes | Yes | Yes | Yes | Yes | Yes | Yes | Yes | Yes | High |
| Rongen-van Dartel et al.2015 | No | Yes | Yes | Yes | Yes | Yes | Yes | Yes | No | Yes | Yes | Yes | Yes | No | No | Yes | Moderate |
| Kelley et al.2018 | No | Partial Yes | Yes | Yes | Yes | Yes | No | Yes | Yes | Yes | Yes | Yes | Yes | No | No | Yes | Moderate |
| Runge et al.2022 | No | Yes | Yes | Partial Yes | Yes | Yes | Yes | Yes | Yes | Yes | Yes | Yes | Yes | Yes | No | No | Moderate |

**Table.2.** Results of assess the methodological quality of meta-analysis
