# Supplementary material for: The effect of exercise therapy on pain, fatigue, bone function and inflammatory biomarkers individuals with rheumatoid arthritis and knee osteoarthritis: a meta-research review of randomized controlled trials
Source: Front Physiol. 2025 Apr 9;16:1558214. doi: 10.3389/fphys.2025.1558214 (PMC12014597; doi:10.3389/fphys.2025.1558214)
Supplement: Supplementary file 3 [file Table3.docx]

**Table 3**: Subgroup analyses for the effects of exercise on health outcomes.

|  | Effect size, *n* | ES (95% CI)^1^ | *I*^2^ (%)^3^ | P-heterogeneity^4^ |
| --- | --- | --- | --- | --- |
| **Pain** |  |  |  |  |
| **Overall** | 14 | -0.50 (-0.87, -0.14) | 83.0 | <0.001 |
| **Duration (min)** |  |  |  |  |
| <30 | 7 | -1.09 (-1.59, -0.60) | 40.7 | 0.119 |
| ≥30 | 7 | -0.04 (-0.30, 0.22) | 62.3 | 0.014 |
| **Type of exercise** |  |  |  |  |
| resistance exercises | 2 | -1.77 (-5.01, 1.47) | 65.2 | 0.102 |
| Aerobic Exercise | 6 | -0.45 (-1.02, 0.11) | 82.6 | <0.001 |
| Aerobic + resistance | 5 | -0.81 (-1.76, 0.14) | 90.2 | <0.001 |
| water-based aerobic | 1 | 0.06 (-0.43, 0.55) | - | - |
| **Age(year)** |  |  |  |  |
| ≤55 | 7 | -0.38 (-0.85, 0.09) | 82.5 | <0.001 |
| >55 | 7 | -0.76 (-1.56, 0.04) | 85.5 | <0.001 |
| **Frequency(week)** |  |  |  |  |
| <3 | 7 | -0.77 (-1.58, 0.03) | 85.9 | <0.001 |
| ≥3 | 7 | -0.39 (-0.86, 0.07) | 81.8 | <0.001 |
| **Type of effect size** |  |  |  |  |
| WMD | 4 | -1.48 (-2.77, -0.18) | 58.6 | 0.065 |
| SMD | 10 | -0.31 (-0.64, 0.02) | 79.7 | <0.001 |
| **Fatigue** |  |  |  |  |
| **Overall** | 3 | -0.28 (-0.44, -0.13) | 55.1 | 0.108 |
| **Type of exercise** |  |  |  |  |
| Aerobic Exercise | 2 | -0.21 (-0.32, -0.09) | 0.0 | 0.877 |
| Aerobic + resistance | 1 | -0.45 (-0.64, -0.25) | - | - |
| **Hand grip** |  |  |  |  |
| Overall | 7 | 0.13 (-0.19, 0.44) | 82.5 | <0.001 |
| **Age(year)** |  |  |  |  |
| ≤55 | 3 | 4.15 (-5.11, 13.40) | 83.8 | <0.001 |
| >55 | 4 | 0.19 (-0.18, 0.57) | 86.3 | <0.001 |
| **duration (min)** |  |  |  |  |
| <30 | 5 | 0.47 (-0.36, 1.30) | 88.2 | <0.001 |
| ≥30 | 2 | -0.08 (-0.21, 0.05) | 0.0 | 0.810 |
| **Type of exercise** |  |  |  |  |
| resistance exercises | 1 | 24.40 (10.30, 38.50) | - | - |
| Aerobic + resistance | 2 | 0.10 (-0.54, 0.75) | 91.4 | <0.001 |
| Aerobic Exercise | 4 | 0.09 (-0.26, 0.44) | 73.1 | 0.011 |
