# Supplementary material for: The effect of exercise therapy on pain, fatigue, bone function and inflammatory biomarkers individuals with rheumatoid arthritis and knee osteoarthritis: a meta-research review of randomized controlled trials
Source: Front Physiol. 2025 Apr 9;16:1558214. doi: 10.3389/fphys.2025.1558214 (PMC12014597; doi:10.3389/fphys.2025.1558214)
Supplement: Supplementary file 4 [file Table4.docx]

**Effect of Exercise on Rheumatoid Arthritis: An Umbrella Review**

**Contents**

[**Supplementary Table 1.** Search strategy to find potential eligible studies 2](#_Toc151241566)

**PubMed**

Number of localized studies: 615

|  | **Descriptors** | **Number of studies reached** |
| --- | --- | --- |
| #1 | "Rheumatic Diseases"[Mesh] OR (Arthritis[Title/Abstract] AND Rheumatoid[Title/Abstract]) OR "rheumatoid arthritis"[Title/Abstract] OR (Arthritis[Title/Abstract] AND Psoriatic[Title/Abstract]) OR "Psoriatic Arthritis"[Title/Abstract] OR "autoimmune rheumatic diseases"[Title/Abstract] OR "rheumatoid arthritis"[Title/Abstract] OR "systemic sclerosis"[Title/Abstract] OR "idiopathic inflammatory myopathies"[Title/Abstract] | 430,401 |
| #2 | exercise[Mesh] OR "physical activity"[Title/Abstract] OR sport[Mesh] OR fitness[Title/Abstract] OR training[Mesh] OR aerobic[Mesh] OR resistance[Title/Abstract] OR "weight loss intervention"[Title/Abstract] OR "interval training"[Title/Abstract] OR "high intensity intermittent exercise"[Title/Abstract] OR HIIT[Title/Abstract] | 2,420,382 |
| #3 | ”Systematic Review" [Publication Type] OR "Meta-Analysis" [Publication Type] | 369,987 |
| #4 | #1 AND #2 AND #3 | 615 |

**Web of Science**

Number of localized studies: 613

|  | **Descriptors** | **Number of studies reached** |
| --- | --- | --- |
| #1 | TS=( (Arthritis AND Rheumatoid) OR “rheumatoid arthritis” OR (Arthritis AND Psoriatic) OR “Psoriatic Arthritis” OR “autoimmune rheumatic diseases” OR “rheumatoid arthritis” ) | 405,320 |
| #2 | TS=(Exercise OR "physical activity" OR sport OR fitness OR training OR aerobic OR resistance OR "weight loss intervention" OR "interval training" OR "high intensity intermittent exercise" OR HIIT) | 5,280,811 |
| #3 | TS=( “systematic review” OR “meta-analysis”) | 578,531 |
| #4 | #1 AND #2 AND #3 | 613 |

**Scopus**

Number of localized studies: 1,373

|  | **Descriptors** | **Number of studies reached** |
| --- | --- | --- |
| #1 | TITLE-ABS-KEY (Arthritis AND Rheumatoid) OR “rheumatoid arthritis” OR (Arthritis AND Psoriatic) OR “Psoriatic Arthritis” OR “autoimmune rheumatic diseases” OR “rheumatoid arthritis”) | 477,973 |
| #2 | TITLE-ABS-KEY (Exercise OR "physical activity" OR sport OR fitness OR training OR aerobic OR resistance OR "weight loss intervention" OR "interval training" OR "high intensity intermittent exercise" OR HIIT) | 6,063,004 |
| #3 | TITLE-ABS-KEY ( “systematic review” OR “meta-analysis”) | 805,261 |
| #4 | #1 AND #2 AND #3 | 1,373 |

**Embase**

Number of localized studies: 871

|  | **Descriptors** | **Number of studies reached** |
| --- | --- | --- |
| #1 | ('Arthritis AND Rheumatoid'/exp OR 'rheumatoid arthritis'/exp OR (Arthritis AND Psoriatic)/exp OR 'Psoriatic Arthritis'/exp OR 'autoimmune rheumatic diseases'/exp OR 'rheumatoid arthritis') | 578,787 |
|  | 'Exercise'/exp OR 'physical activity'/exp OR 'sport'/exp OR 'fitness'/exp OR 'training'/exp OR 'aerobic'/exp OR 'resistance'/exp OR 'weight loss intervention'/exp OR 'interval training'/exp OR 'high intensity intermittent exercise'/exp OR 'HIIT'/exp | 1,317,663 |
| #2 | 'systematic review'/exp OR 'meta-analysis'/exp | 649,548 |
| #3 | #1 AND #2 AND #3 | 871 |

**Cochrane**

Number of localized studies: 38

Limits: TRIALS

|  | **Descriptors** | **Number of studies reached** |
| --- | --- | --- |
| #1 | (Arthritis AND Rheumatoid) OR “rheumatoid arthritis” OR (Arthritis AND Psoriatic) OR “Psoriatic Arthritis” OR “autoimmune rheumatic diseases” OR “rheumatoid arthritis”):ti,ab,kw | 31711 |
| #2 | (Exercise OR "Physical Activity" OR sport OR fitness OR training OR aerobic OR resistance OR "weight loss intervention" OR "interval training" OR "high intensity intermittent exercise" OR HIIT ):ti,ab,kw | 370,650 |
| #3 | #1 AND #2 | 38 |

**Supplementary figure 1. Funnel plot of pain
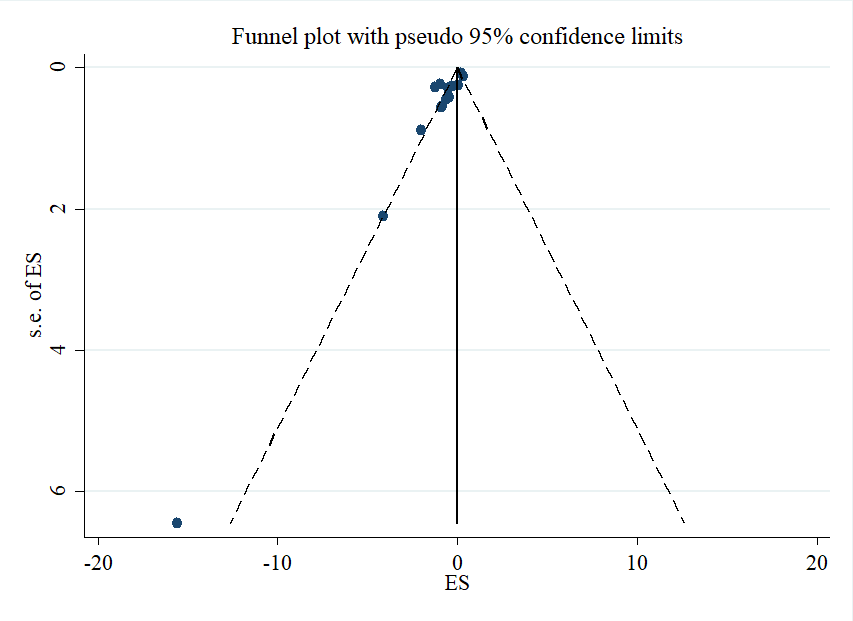
**
